# Addiction of MYCN Amplified Tumours to B-MYB Underscores a Reciprocal Regulatory Loop

**DOI:** 10.18632/oncotarget.138

**Published:** 2010-08-04

**Authors:** Francesco Gualdrini, Daisy Corvetta, Sandra Cantilena, Olesya Chayka, Barbara Tanno, Giuseppe Raschellà, Arturo Sala

**Affiliations:** ^1^Molecular Haeamatology and Cancer Biology Unit, UCL Institute of Child Health, London WC1N 1EH, UK; ^2^ENEA Research Center, Laboratory of Radiation Biology and Biomedicine Via Anguillarese, 301, 00123 S. Maria di Galeria, Rome, Italy

**Keywords:** neuroblastoma, oncogene, synthetic lethality, transcription factor

## Abstract

MYCN is a member of the MYC family of oncoproteins frequently amplified or overexpressed in aggressive, paediatric tumours of the nervous system. In this study we have identified the gene B-MYB, encoding the transcription factor also known as MYBL2, as a downstream target of MYCN. Using multiple *in silico* databases we show that expression of B-MYB significantly correlates with that of MYCN in neuroblastoma patients. MYCN binds to and activates the B-MYB gene *in vivo* and *in vitro*. Blunting B-MYB expression by RNA interference causes reduced proliferation of MYCN amplified, but not MYCN-non amplified, neuroblastoma cell lines, indicating that tumour cells are addicted to B-MYB in a MYCN dependent manner. Notably, B-MYB binds in vivo to the MYCN amplicon and is required for its expression. We conclude that MYCN and B-MYB are engaged in a reciprocal regulatory loop whose pharmacological targeting could be beneficial to patients with the aggressive forms of cancer in which MYCN is amplified.

## INTRODUCTION

Neuroblastoma is the most frequent extracranial solid cancer in childhood and is formed by proliferating precursor cells of the sympathetic nervous system that fail to terminally differentiate [1-3]. Neuroblastoma’s behaviour range from spontaneous regression to an infaust outcome [1]. In 1983 Schwab and colleagues identified a MYC-related oncogene, named MYCN, that was amplified in a panel of neuroblastoma cell lines [4]. Amplification of MYCN is detected in 20-30% of neuroblastomas and is the most important genetic aberration in this disease, strongly related to poor outcome [5]. MYCN is expressed in the migrating neural crest and encodes a protein with a basic helix-loop-helix [bHLH] domain that takes part in the Max network, with a crucial role in governing cell growth, apoptosis, and differentiation [6]. Transcription modulation of target genes is carried out by the hetero-dimer Myc/Max which binds to the E-box consensus CA(C/T)GTG in target genes promoters with high efficiency [7]. Several studies established the strict link between MYCN amplification/over-expression and the transformed phenotype [8,9], but it is still unclear what are the key cellular genes regulated by MYCN involved in its transforming ability. Furthermore, whether there are cellular factors limiting for the expression of the MYCN amplicon in neuroblastoma cells has not been investigated in previous studies.

B-MYB is a transcription factor of the MYB family associated with advanced neuroblastoma stages and whose over-expression confers drug resistance to neuroblastoma cells [10,11]. B-MYB is key for proliferation and survival of mammalian cells and embryonic development in mice [12]. B-MYB is thought to promote cell proliferation cooperating with factors important for the cell cycle, like E2Fs, or required for cell division, like cdc2, cyclin A2 and cyclin B1 [13-15]. Promotion of cell survival by B-MYB could be achieved by transactivation of pro-survival genes such as BCL2 [16,17]. Similar to other oncogenic transcription factors, B-MYB is broadly over-expressed or amplified in different types of human cancer including breast, lung, ovary carcinomas [18-22]. We have previously shown that B-MYB is associated to the risk of developing neuroblastoma, its expression is increased in patients with metastatic disease and predicts poor survival [11,23].

In this study, we monitored the expression of B-MYB and MYCN in multiple microarray experiments containing data from hundreds of neuroblastoma patients. This analysis revealed that B-MYB is significantly co-expressed with MYCN in neuroblastoma patients and in the follow-up experiments we show that MYCN and B-MYB regulate each other with important biological consequences.

**Figure 1:**
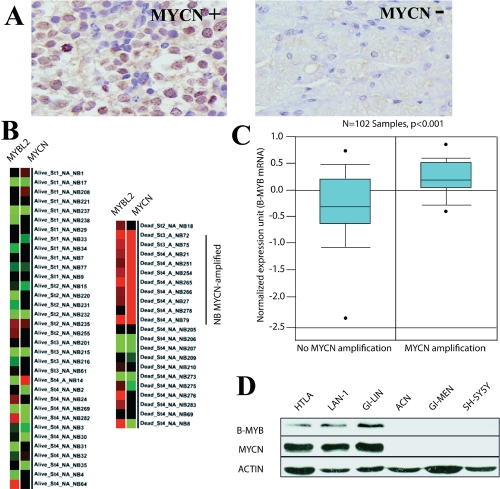
Expression of B-MYB correlates with MYCN status in neuroblastomas. A) Immunohistochemical analysis of B-MYB protein in primary human neuroblastomas. Strong nuclear B-MYB staining is detected in the MYCN amplified specimens [n = 3]. B) B-MYB gene expression profiling in 56 pretreatment primary neuroblastoma tumor samples. For each sample is specified the stage, the outcome (alive/dead) and the presence of MYCN amplification. Red indicates high whereas green indicates low gene expression levels. The expression profile was mined from the Oncogenomics web site (home.ccr.cancer.gov/oncology/oncogenomics/) C) Box plot of B-MYB mRNA epression in primary human neuroblastomas with or without MYCN amplification. The plot was obtained using the Oncomine Web site (www.oncomine.org), where the information regarding the neuroblastoma samples can also be found. Statistical significance was assessed by Student *t* test [p < 0.001 in 102 samples]. mRNA datasets are normalized by being log 2 - transformed, with the median set to 0 and SD set to 1. All statistical tests were two-sided. C) Western blot analysis showing expression of B-MYB in MYCN amplified or non amplified cell lines. Antibodies recognising MYCN, B-MYB and actin [as a loading control] were used, as indicated.

## RESULTS

### B-MYB and MYCN are co-expressed in neuroblastoma tumours and cell lines

We assessed 24 primary neuroblastoma specimens by immunohistochemistry to study the expression and subcellular localisation of the B-MYB protein. Nuclear staining of B-MYB was frequently detected (54% of samples) in neuroblastomas and expression was particularly strong in MYCN amplified cases (Fig 1A). To investigate whether the expression of B-MYB and MYCN is statistically significantly correlated in neuroblastoma patients, we have used three databases: Genesapiens [24] [www.genesapiens.org], Oncomine [25] [www.oncomine.org] and Oncogenomics [26] [home.ccr.cancer.gov/oncology/oncogenomics/]. We firstly confirmed that the B-MYB mRNA is highly expressed in neuroblastoma tumours and its levels, in agreement with previous findings [11], correlate with poor patients’ survival (data not shomn). Interestingly, we observed that in all the available databases the expression of B-MYB is statistically significantly higher in specimens in which the MYCN proto-oncogene is amplified (Figure 1B,C and data not shown).

To verify the correlation between MYCN and B-MYB at the protein level, we carried out western blot analysis of B-MYB and MYCN expression in MYCN amplified and non amplified neuroblastoma cell lines. As expected, neuroblastoma cell lines with amplification of MYCN also over-express B-MYB, validating the microarray data (Fig 1D).

### MYCN binds to and transactivates the B-MYB gene

**Figure 2:**
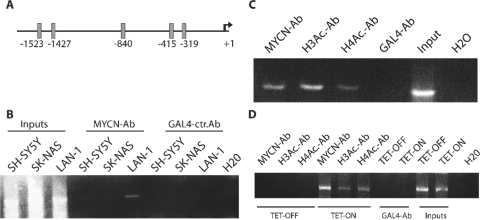
MYCN binds to the B-MYB promoter *in vivo* and induces histones acetylation. A) distribution of the five putative E-BOXes on the B-MYB promoter found by the TESS analyzer tool [www.cbil.upenn.edu/cgi-bin/tess/tess]. B) Chromatin IP analysis of the MYCN protein bound onto the B-MYB promoter in SHSY5Y and SKNAS (non MYCN amplified) and LAN-1(MYCN amplified) neuro-blastoma cell lines. Cross-linked chromatin was immuno-precipitated with MYCN or GAL4, used as a negative control, antibodies as indicated. C) Chromatin-IP analysis to assess the presence of acetylated histones H3 and H4 on the B-MYB promoter region containing the putative MYCN binding sites. The antibodies used are indicated on the top of the figure. D) Chromatin-IP analysis of factors bound onto the B-MYB promoter in the presence or absence of inducible MYCN. Antibodies used and conditions are shown in the top of the panel.

We sought to determine whether MYCN could directly regulate B-MYB. To verify the presence of MYC binding sequences in the B-MYB promoter, we used the TESS Web tool (www.cbil.upenn.edu/cgi-bin/tess/tess) [27]. There are 5 canonical and non canonical E-BOXes in the B-MYB promoter at position −1523 (CACCTG), −1427 (CACGTG), −840 (CACCGT), −415 (CACGTG), −319 (CAGGTG) (Figure 2A).

Analysis of the distribution of the MYC binding sites shows that each sequence is separated from one another by a distance in base pairs multiple of 140bp, necessary for a double turn of the DNA around the nucleosome. This means that all the E-BOXs are exposed on the same side of the DNA helix. Furthermore the distance between two of the E-BOXes (−1523/−1427 and −415/−319) is around 100bp. This distance is required for the formation of an anti-parallel tetramer between two MYCN-MAX heterodimers [28].

To verify that MYCN could interact with the B-MYB promoter region encompassing the putative E-BOXes in human neuroblastoma cells *in vivo*, we carried out Chromatin-IP analysis, which confirmed that MYCN binds to the B-MYB promoter only in a MYCN-amplified cell line (Figure 2B). This region of the B-MYB promoter bound by MYCN contains acetylated histones H3 and H4, suggesting that the chromatin is in a relaxed, active state (Figure 2C).

To investigate whether histone acetylation was a direct effect of MYCN binding, we used a cell line which carry a MYCN cDNA under the control of a tetracycline-inducible promoter. As expected, no binding of MYCN onto the B-MYB promoter was observed in uninduced cells, and acetylation of histones H3 and H4 was only observed after binding of MYCN on the promoter (Fig 2D).

**Figure 3:**
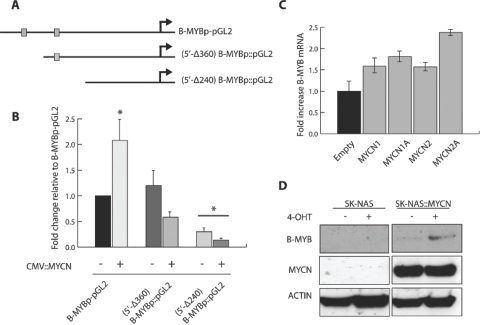
MYCN transcriptionally activates B-MYB MRNA and protein. A) Schematic representation of three reporter vectors containing fragments of the B-MYB promoter: B-MYBp-pGL2 (from position −102 to −709, relative to the transcription start site); 5’-Δ360 (from position −102 to position −360); 5’-Δ240 (from position −102 to −240). B) Quantifications of luciferase assays showing the activity of the B-MYB promoter segments in the presence or absence of exogenously expressed MYCN. Error bars indicate standard deviations and the asterisk indicates statistically significant differences [* = p < 0.001] with respect to the levels of B-MYBp-pGL2 promoter activity, which was arbitrarily set to 1. C) Real time PCR analysis of B-MYB expression in 293 cells transiently transfected with a MYCN expression vector. The expression levels of four independent MYCN transfectants relative to cells transfected with empty vector are shown. Error bars indicate standard deviations obtained from triplicate assays. D) Western blot analysis showing enhanced expression of B-MYB caused by activation of MYCN in SKANAS::MYCN(ER) cells. (+ or −) 4-OHT indicates that the cells were cultured in the presence or absence of 4-hydroxytamoxifen, respectively.

To study the transcriptional regulation of B-MYB in human NB cells, we used a B-MYB promoter construct linked to the luciferase gene, containing the two E-BOXes most proximal to the transcription start site, including the canonical −415 E-BOX, or two additional constructs in which these E-BOXes are progressively deleted (Figure 3A). MYCN over-expression caused a two fold increase in luciferase activity only in the presence of both the E-BOXes. Deletion of the distal, canonical −415 E-BOX sequence was sufficient to completely abrogate MYCN transactivation, suggesting that binding of MYCN to this promoter region is functionally relevant Figure 3B).

We next assessed whether MYCN could activate endogenous B-MYB in human cell lines. Firstly, MYCN was transiently transfected into H293 cells and the expression of the B-MYB mRNA was quantified by Q-PCR. We observed a 1.56- to 2.38-fold increase in B-MYB expression levels after MYCN transfection (Figure 3C), Secondly, we used a neuroblastoma cell line containing a construct conditionally activating MYCN in the presence of 4-hydroxytamoxifen. After activation of MYCN, western blot analysis showed that B-MYB is induced at the protein level, validating the hypothesis that B-MYB is under the control of MYCN in human cancer cell lines (Figure 3D).

### B-MYB is essentially required for the proliferation of MYCN-amplified neuroblastoma cell lines

**Figure 4:**
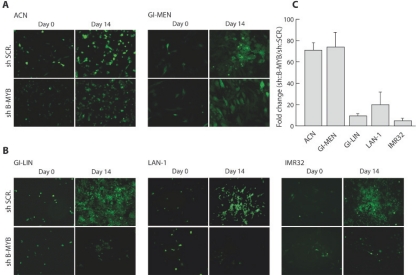
B-MYB is required for proliferation of MYCN amplified cell lines. A) Proliferation assay of non MYCN amplified GIMEN and ACN cells infected with a lentiviruses expressing a scrambled or B-MYB shRNA, and selected with puromycin for 14 days. B) Proliferation assay of MYCN amplified GI-LIN, LAN-1 and IMR32 cells infected with lentiviruses expressing a scrambled or B-MYB shRNA, and selected with puromicin for 14 days. C) Quantification of the proliferation assays. The error bars indicate the standard deviations of the values obtained in 3 replicate infections. The experiment was repeated twice with similar results.

To determine whether expression of B-MYB is required for proliferation of MYCN-amplified neuroblastoma cells, three MYCN-amplified (LAN-1, GI-LIN, IMR-32) and two MYCN non amplified (GI-MEN, ACN) cell lines were infected with lentiviruses carrying B-MYB, or scrambled shRNAs, and expressing GFP. After infections, cells were quickly selected with puromycin and GFP positive cells were counted and re-plated in 60mm dishes at low density. After 14 days, knock-down of B-MYB caused inhibition of proliferation of LAN-1, GI-LIN and IMR-32, but not GI-MEN and ACN, neuroblastoma cells (Figure 4A,B and C). The lack of phenotypic effect in the non-MYCN amplified cell lines occurred despite the shRNA vector caused a marked downregulation of B-MYB protein expression (Figure S1).

The B-MYB shRNA caused reduced cell cycle activity of LAN1 cells, which showed a block in G1, while IMR-32 cells were not affected (Figure S2), suggesting that the negative effect of B-MYB depletion on cell proliferation can be caused by reduced cell cycle activity or increased cell death. Indeed, we observed a marked increase of IMR-32 cells detaching from the dish during selection of cells infected with the B-MYB shRNA lentivirus, compared to the control virus-infected cells. These observations are in keeping with the hypothesis that B-MYB is downstream of MYCN since down-regulation of MYCN in neuroblastoma cells was previously shown to cause cell cycle arrest or apoptosis [29,30].

### B-MYB binds to and regulates the MYCN promoter

**Figure 5:**
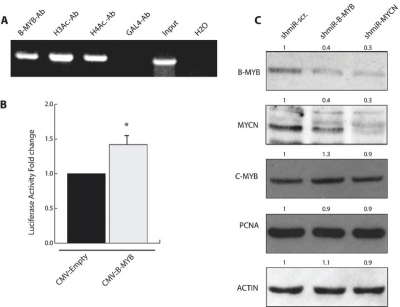
B-MYB binds to and regulates the MYCN promoter. A) Chromatin-IP analysis of proteins bound onto the MYCN promoter region encompassing the putative MYB-binding sites. Antibodies to immunoprecipitate B-MYB, acetylated histones H3 and H4 or GAL4 (used as a negative control) in LAN-1 [MYCN amplified] cells were used, as indicated in the top of the panel. B) Luciferase assay to assess MYCN promoter (MYCNp-pGL2) transcriptional activity in the presence or absence of exogenously expressed B-MYB. This assay was repeated several times until it reached statistical significance [*p < 0.01] C] Western blot analysis of IMR32 cell lines infected with lentiviruses expressing, in order from left to right, sh::scrambled, sh::B-MYB and sh::MYCN, and selected with puromycin for 6 days. Whole cell lysates were prepared and subjected to western blot analysis with the indicated antibodies. Relative intensity of the bands are indicated on the top of the lanes.

c-MYB and B-MYB regulate the expression of the prototype member of the MYC family, c-MYC [31]. We then speculated that B-MYB could be involved in the regulation of MYCN in the context of neuroblastoma. We firstly investigated whether MYB binding sites are present in the MYCN promoter. Analysis of about 2Kb of the 5’ flanking region of the MYCN gene revealed that there are 7 putative MYB binding sites. To asses whether B-MYB binds the MYCN promoter *in vivo*, we performed Chromatin-IP assays on chromatin extracted from LAN-1 cells. We found that B-MYB is tightly bound to the MYCN promoter and the same chromatin domain contains acetylated histones, consistent with the idea that this segment of the MYCN promoter is active (Figure 5A). Next, we subcloned the MYCN promoter segment containing the MYB binding sites into the luciferase reporter vector PGL2 and investigated whether this was activated by B-MYB. We observed a weak but reproducible increase of MYCN promoter activity in the presence of exogenous B-MYB, suggesting that its interaction with the MYCN amplicon is functionally relevant (see discussion) (Figure 5B).

To investigate whether a reciprocal feedback loop is operating in human neuroblastoma cells with amplification of MYCN, we infected IMR-32 cells with B-MYB, or scrambled, shRNA lentivirus. After puromicyn selection, the expression of B-MYB and MYCN was assessed by western blot analysis. We observed that knockdown of B-MYB affected the expression of MYCN and *vice versa*, suggesting that MYCN and B-MYB require each other for their expression (Figure 5C). Notably, the expression of c-MYB and the proliferation marker PCNA were unchanged by the knockdown of B-MYB or MYCN, demonstrating that their reciprocal regulation is specific and not a consequence of inhibition of cell proliferation.

### Exogenous overexpression of MYCN does not promote addiction to B-MYB in neuroblastoma cells

Addiction of MYCN amplified cell lines to B-MYB could be explained by two theories: a) B-MYB is required for expression of the MYCN amplicon and addiction of neuroblastoma cells to B-MYB reflects the dependence of amplified cell lines to MYCN expression; b) B-MYB is downstream of MYCN signalling and/or required to overcome senescence/apoptotic signals triggered by MYCN overexpression.

**Figure 6.**
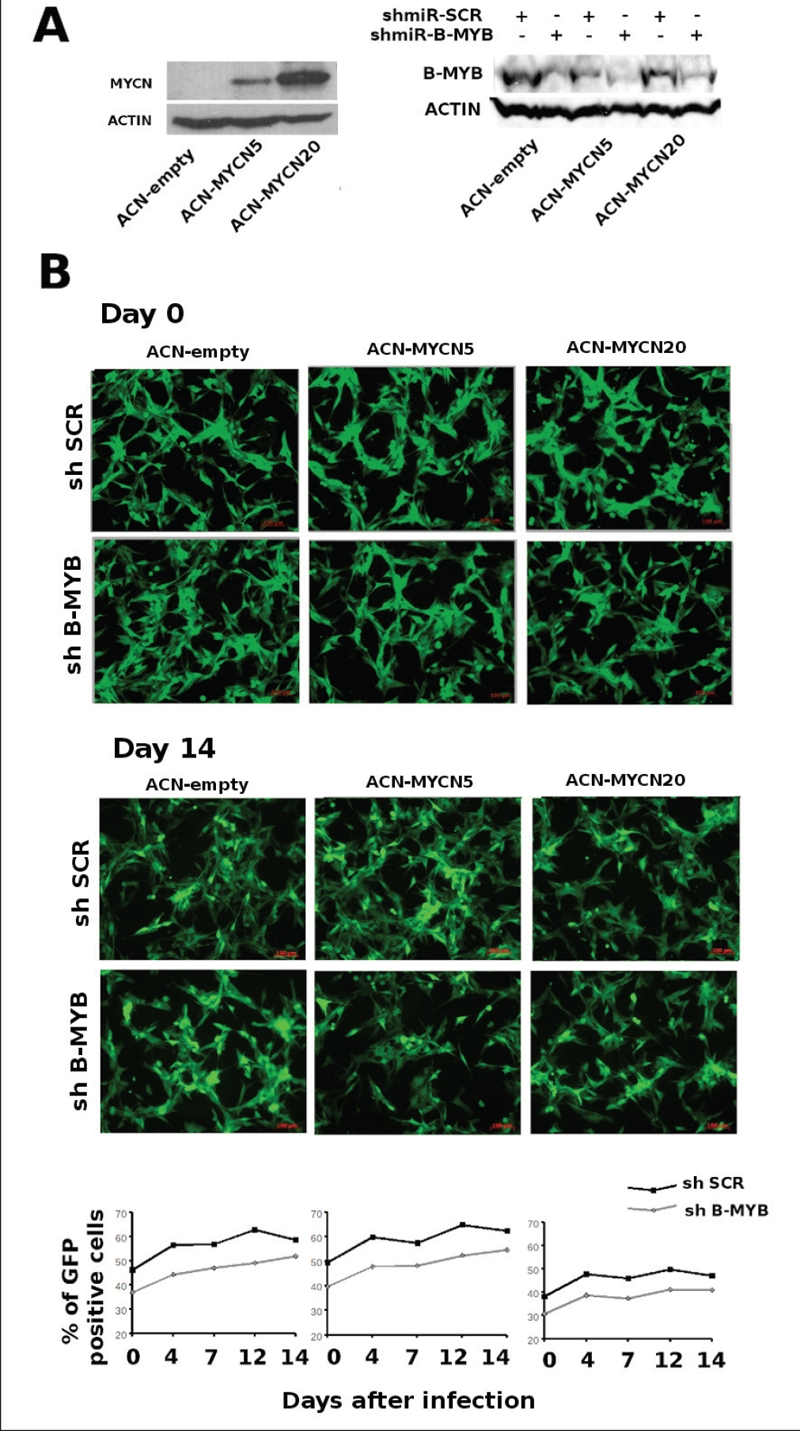
Exogenous expression of MYCN does not induce addiction of neuroblastoma cells to B-MYB. A) left panel: western blot analysis showing the expression of MYCN in ACN clones stably transfected with different dosages of the MYCN, or control, plasmids. Right panel: western blot analysis to detect the expression of B-MYB in the ACN/MYCN clones infected with B-MYB, or scrambled control, shRNA viruses. B) Proliferation assay of the MYCN, or control, ACN clones infected with the scrambled or B-MYB shRNA lentiviruses. Quantification of the percentages of GFP positive (infected) cells at the indicated days shows that proliferation of the different cell lines is not affected by depletion of B-MYB. Please note that in this assay cells were not counted and plated equally at the start of the experiment and the different percentages of GFP positivity at time 0 reflect variable infection efficiency.

To distinguish between these two possibilities, we stably transfected the non MYCN amplified cell line ACN with an expression vector containing the MYCN cDNA under the control of the CMV promoter. We then assessed whether ablating B-MYB expression by RNA interference caused a phenotypic effect in two independent MYCN expressing clones. As opposed to naturally amplified cell lines, the MYCN-transfected clones continued to proliferate normally in spite of reduced expression of B-MYB (Figure 6). Thus, we conclude that addiction of neuroblastoma cells to B-MYB is contingent on the presence of the MYCN amplicon and reflects the crucial role of B-MYB in its transcriptional regulation.

## DISCUSSION

Neuroblastoma is a challenging disease for clinicians, with an enigmatic and complex biology [1]. There are several cyctogenetic aberrations in neuroblastoma but, by far, the most clinically relevant is the amplification of MYCN. MYCN amplification is associated with metastatic disease and poor survival of neuroblastoma patients. Inhibition of MYCN by antisense or RNA interference approaches decreases the G1-S transition of the cell cycle or induces apoptosis in MYCN amplified neuroblastoma cells in culture [30,32]. Furthermore, in the absence of MYCN, NE2F and ID2 expression is decreased whereas there is an increase of the growth suppressor p27 [29]. Transgenic expression of MYCN in the neuroectoderm causes neuroblastoma in mice, demonstrating its causative role in this disease [8]. In principle, MYCN could be an ideal target for therapy: it is aberrantly overexpressed in neuroblastoma and only marginally present in normal tissues. Apart from the intrinsic difficulty of targeting a transcription factor, the development of MYCN-targeting drugs is hampered by its extremely high expression in tumour cells. In principle, it would be better to direct clinical intervention towards genes upstream or downstream of the MYCN pathway, more amenable to drug inhibition.

The product of the B-MYB [MYBL2] gene, is a transcription factor that modulates the cell cycle through activation of critical regulators of cell division, also required for cell survival. Aberrant B-MYB expression or amplification has been documented in different types of human cancers, suggesting a role in tumorigenesis [33-35]. Furthermore B-MYB was recently implicated in the maintenance of pluripotency, chromatin stability and normal cell cycle progression [36-38].

There is some evidence that B-MYB might have a role in neuroblastoma. For example, we have shown previously that the expression of B-MYB stratifies patients with aggressive forms of the disease and specific variants of B-MYB are associated with an increased risk of developing neuroblastoma [11,23]. Furthermore, B-MYB is aberrantly stabilised in neuroblastoma cell lines, increasing its pro-survival function [39]. The Ishii laboratory was the first to show a relationship between MYC and MYB transcription factors by reporting that c-MYB and B-MYB are able to bind to and transactivate c-MYC promoter segments in gel shifts and transient transfection assays, respectively [31]. In the light of this study, we asked whether MYCN and B-MYB were functionally linked in neuroblastoma and, if so, what are the functional consequences. We initially observed that MYCN and B-MYB are co-expressed in neuroblastoma patients with aggressive or fatal disease (Figure 1A). We then went on to show that B-MYB expression is selectively required for proliferation of MYCN amplified tumours unveiling a reciprocal regulatory loop of the two transcription factors in neuroblastoma.

**Figure 7.**
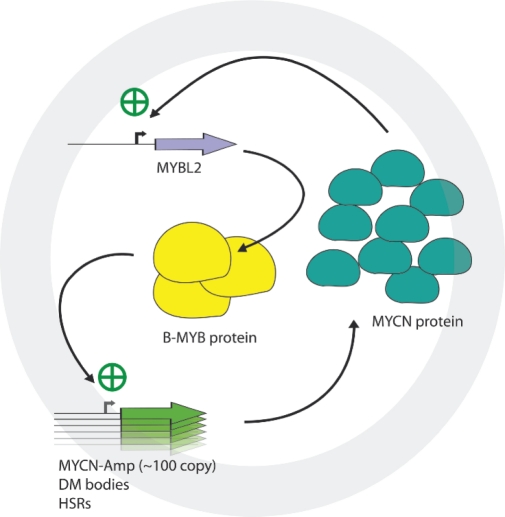
Schematic representation of the feedback loop governing B-MYB and MYCN expression in MYCN amplified cell lines.

A number of findings described in this study have important biological and clinical significance. Firstly, B-MYB depletion causes synthetic lethality in MYCN amplified cell lines, demonstrating its key role in the regulation of the MYCN amplicon. B-MYB is a relatively weak, ubiquitous transcription factor and we speculate that in normal physiological settings it is not essential for the transcription of MYCN, whose expression is strictly tissue specific. On the other hand, MYCN could be involved in the expression of B-MYB during neurogenesis. Indeed, B-MYB is expressed under the control of neuronal signalling molecules like MELK and it is required for proliferation and survival of progenitor cells in the developing nervous system [40]. One likely scenario is that in tumours of the nervous system like neuroblastoma, the generation of multiple copies of the MYCN gene in DM bodies or HSRs chromosomes causes accumulation of the MYCN oncoprotein which binds to the B-MYB locus, activating its unregulated expression. This, in turn, will initiate a pathological regulatory cycle where B-MYB, in spite of its intrinsically weak transcriptional activity (Figure 5B), will cause a significant enhancement of MYCN expression due to the large number of amplicons available (see model in Figure 7). This theory should explain the apparent paradox of a ubiquitous factor B-MYB-being upstream of a tissue specific factor –MYCN- and clarify why MYCN is not activated in the vast cohort of non-neuronal tumours where B-MYB is overexpressed or amplified.

Our work has potential clinical implications. Given their reliance on B-MYB, tumours with amplification of MYCN should be exquisitely sensitive to its pharmacological targeting, indicating that the search for small molecule inhibitors of B-MYB is warranted.

## MATERIALS AND METHOD

### Patient samples and immunohistochemistry

The use of tumour samples was authorized by the National Research Ethics Service (NRES), REC reference number 10/H0706/19. Patient archival diagnostic samples were identified by searching the histopathology database of a single institution (Great Ormond Street Hospital) using the search criteria neuroblastoma or ganglioneuroblastoma and identifying cases diagnosed between August 1994 and August 2005. Amplification of MYCN was identified by real time PCR.

Sections (4mm) from formalin-fixed paraffin embedded samples (24 patients) were cut and mounted onto slides, which were treated with xylene and graded alcohol, and equilibrated in PBS. Antigen retrieval was performed by microwaving for 5 min (three times at 400W) in a buffer containing 10mM sodium citrate pH 6. Of the 24 patients analysed, 13 were positive for nuclear B-MYB (54%). Among the positives samples, 3 were MYCN amplified. None of the B-MYB negative samples were MYCN amplified.

### Antibodies

Monoclonal anti-B-MYB [41], was kindly provided by Roger Watson. Other primary antibodies used were: anti-MYCN, anti-B-MYB, anti-Actin (Santa Cruz Biotechnology), anti-acethyl histone H3 and H4 (Upstate). Horseradish peroxidase-conjugated secondary antibodies were purchased from GE Healthcare Life Sciences.

### Plasmids

652 base pairs of the MYCN promoter (from position −32 to −684) were cloned into the pGL2-basic vector (Promega) and were obtained by amplifying this region from human genomic DNA using the following primers: FW-5’AACTCGAGGAGGGGAAGGATTTGTG 3’ Rev- 5’ATTAAGCTTCTTTCCGCCCCGTTCG 3’. The PCR product was cloned into the topo-vector (Invitrogen) and sub-cloned again into the pgl2-basic (Promega) using XhoI and HindIII restriction sites. The pGL2 constructs containing segments of the B-MYB promoter were described previously [42]. CMV-BMYB and CMV-MYCN we also described in previous studies [23,43]. Trans-Lentiviral™ GIPZ vectors coding for three types of shRNA::B-MYB (V2LHS_152045, V2LHS_152043, V2LHS_263085), three for MYCN (V2LHS_4509, V2LHS_36751, V2LHS_36755) and one scrambled pGIPZ (RHS_4346) were from Open Biosystem.

### Cell lines

LAN-1, IMR-32 and HEK293T cell lines were obtained from ATCC and maintained in DMEM media (Invitrogen) supplemented with 10% FBS (Invitrogen), penicillin, streptomycin, sodium pyruvate and non essential amino acid. ACN, GI-LIN, GI-MEN were a kind gift of Mirco Ponzoni (Gaslini Hospital, Genova, Italy), SK-NAS::MYCN[ER] cell lines were a gift from Mike Hogarty (University of Pennsylvania, Philadelphia, USA) and SHSY-5Y cells conditionally expressing MYCN under the control of a tetracycline responsive promoter were a gift from Giovanni Perini (University of Bologna, Italy). Neuroblastoma cell lines were authenticated by assessing the expression of MYCN and the neuronal marker CD56.

### Transfections And Luciferase Report Assay

Transfections were carried out with Lipofectamine 2000 following the manufacturer’s protocol (Invitrogen). Luciferase activity was assessed with the luciferase Assay Kit (Promega).

### Real-time Pcr

Quantitative real-time PCR was carried out with the Taq-man Master Mix (Applied Biosystems) using the ABI-PRISM 7000 Sequence Detection System. Primers and probes were purchased from Applied Biosystems. The expression of each gene was normalized using GADPH as a reference, and relative levels were quantified by calculating 2−ΔΔCt, where the ΔΔCt is the difference in Ct between target and reference.

### Western blot Analysis

Cells were lysates in RIPA Buffer (10 mM Tris-Cl [pH 8.0], 1 mM EDTA, 1% Triton X-100, 0.1% sodium deoxycholate, 0.1% SDS, 140 mM NaCl) and protease inhibitors (Roche). Proteins were separated by SDS/PAGE on 10% gels, transferred to poly vinylidene difluoride membrane (Amersham Pharmacia), and incubated with antibodies. Immunoblots were visualized by using the enhanced chemiluminescent system (Thermo).

### Chromatin Immunoprecipitation (Chip) And Gel Shift

For ChIP assays, after fixing the protein/DNA complexes with formaldehyde we prepared lysates from 1.0 × 107 log-phase neuroblastoma cells essentially as described by the manufacturer’s protocol (ActiveMotif’s ChIP-IT Express kit). Immunoprecipitated chromatin was amplified using the following primers pairs: B-MYB promoter, forward, 5′-GGACCCAGTAGTGGCTTGGA-3′; reverse, 5′-CGCTACTTCGGAGTTGTGGA-3′. MYCN promoter: forward, 5′-CCCTGCTATCATTTGCACTC-3′; reverse 5′-CGTTTTAATACCGGGGGTGC-3’. Gel shift analysis was carried out as described previously [10]. The oligonucleotides used to generate the double straneded probes were:

Wild type E-box:

5’-CCCGGACTGACACGTGAGCCAGGCCTCG-3’

Mutant E-box:

5’ CCCGGACTGATACCTGAGCCAGGCCTCG 3’

### Lentiviral Constructs And Infections

For lentiviral shRNA expression, viral particles were produced in HEK-293T cells transfected with appropriate packaging plasmids. pGIPZ lentiviral vector was obtained from Open Biosystems, Surrey, UK. The non-silencing shRNAmir construct (scrambled shRNA) served as the negative control. 24 hrs after transfections, supernatants were collected, supplemented with 8 mg/ml of polybrene (Sigma) and filtered through a 0.45-mm filter unit. Target cells were infected for 24-h at 37 °C. To asses the effect of shRNA lentiviruses on cell proliferation, cells were and rapidly selected with puromycin (1-5 μg/ml) for 3-4 days. GFP positive cells (0.3x10^6 cell in 60mm dish) were plated and counted after 14 days.

### Cell Cycle Analysis

Cells were fixed on ice with 70% ethanol, then washed and resuspended in PBS containing 2mg/ml Propidium Iodide, 0.1% NP-40 and RNAse followed by FACS analysis.

## SUPPLEMENTAL FIGURE

Supplemental Figure 1
