# Supplementary material for: Addiction of MYCN Amplified Tumours to B-MYB Underscores a Reciprocal Regulatory Loop
Source: Oncotarget. 2010 Aug 4;1(4):278–88. doi: 10.18632/oncotarget.138 (PMC3248110; doi:10.18632/oncotarget.138)
Supplement: Supplemental Figure 1 [file oncotarget-01-278-s001.pdf]

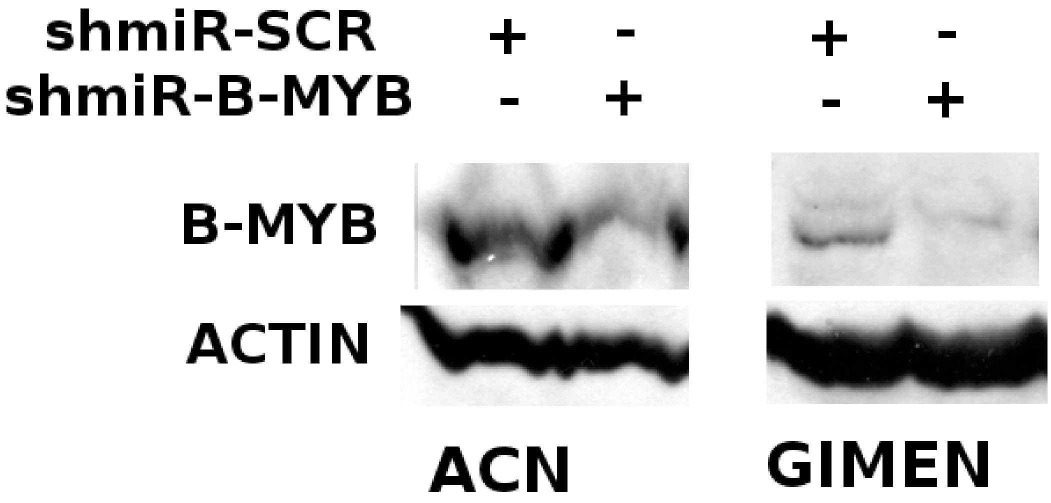

Figure S1. Western blot analysis to assess the expression levels of B-MYB in the non MYCN amplified Gimen and ACN cell lines. Expression of Actin was used as a loading control.

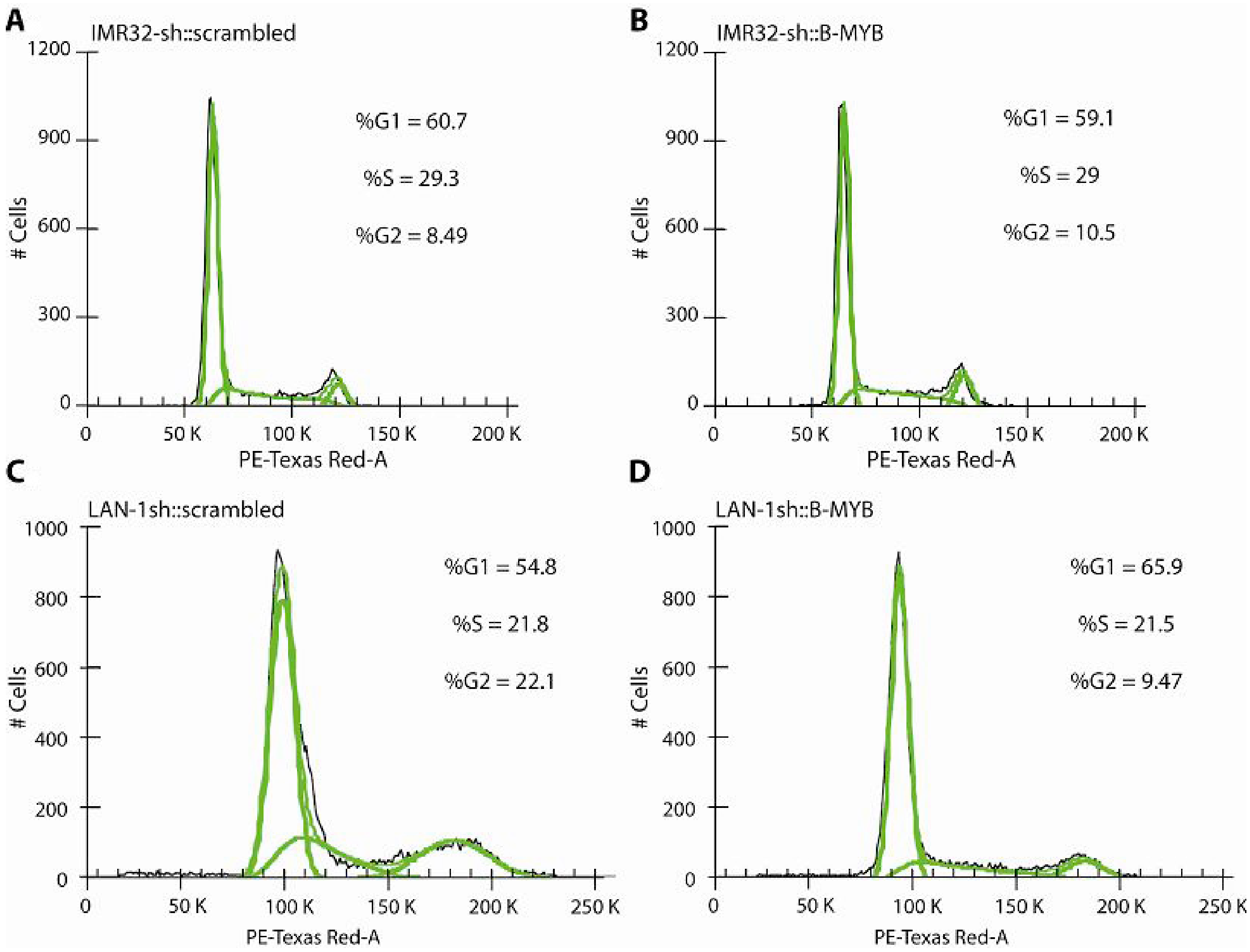

Figure S2. Cell cycle profile of IMR32 and LAN-1 cell lines infected with lentiviruses expressing B-MYB or scrambled shRNAs. IMR32 or LAN1 cells were infected with sh-scrambled (panels A and C) or sh-B-MYB (panels B and D) lentiviruses, selected with puromycin for 14 days and fixed for FACS analysis. The graph shows the distribution of cells in G1, S and G2. It was not possible to assess apoptosis since dead cells were removed during the selection process.
